# Supplementary material for: Gene regulatory network integration with multi-omics data enhances survival predictions in cancer
Source: Brief Bioinform. 2025 Jul 7;26(4):bbaf315. doi: 10.1093/bib/bbaf315 (PMC12229988; doi:10.1093/bib/bbaf315)
Supplement: Supplementary_material_bbaf315 [file supplementary_material_bbaf315.pdf]

Supplementary material

Supplementary methods

Mapping factor weights to omics features when using PCA prior to JDR

In order to perform downstream analysis with the MOFA factors, we needed to map the MOFA factors back to the original features (such as genes). We approximated this mapping as:

$$W_{\text{MOFA}} \approx P_{\text{filtered}} W_{\text{PC-fil}} \quad (1)$$

Here, W_{MOFA} is the matrix of MOFA weights for one omic dataset, P_{filtered} is the matrix of PCA loadings filtered based on a cumulative R^2 threshold (see Section 2.4 in the main manuscript), and $W_{\text{PC-fil}}$ is the matrix of MOFA weights for the filtered principal components of the omic dataset. Below is the mathematical justification.

Given an omic matrix X, PCA finds a matrix of PCA scores S_{PCA} as given by (2) [1]. P is the matrix of PCA loadings.

$$S_{\text{PCA}} = XP \quad (2)$$

From (2), we can express X as (see relationship between PCA and single value decomposition [1, 2]):

$$X = S_{\text{PCA}} P^T \quad (3)$$

Let's now consider the matrix factorization performed by MOFA. Given Q omic matrices Y_i, for $i = 1, \ldots, Q$, MOFA implements a linear factorization of the omic matrices as given by (4) [3, 4].

$$Y_i = W_{\text{MOFA},i} F_{\text{MOFA}} + E_{\text{MOFA},i}, \text{ for } i = 1, \ldots, Q \quad (4)$$

Where F_{MOFA} is a factor matrix common to all omics, and $E_{\text{MOFA},i}$ is the error or residual noise. For simplicity, we will focus here on only one omic matrix Y.

$$Y = W_{\text{MOFA}} F_{\text{MOFA}} + E_{\text{MOFA}} \quad (5)$$

One important note is that PCA and MOFA expect input in different formats. PCA expects input to be formatted as samples $\times$ features, whereas MOFA expects input with features $\times$ samples format. Thus:

$$Y = X^T \quad (6)$$

From (3) and (6):

$$Y = PS_{\text{PCA}}^T \tag{7}$$

In our analysis, we input the transposed PCA score matrix $S_{\text{PCA},i}^T$ into MOFA instead of the original omic matrix Y.

$$S_{\text{PCA}}^T = W_{\text{PC}}F_{\text{PC}} + E_{\text{PC}} \tag{8}$$

From (7) and (8):

$$Y = P(W_{\text{PC}}F_{\text{PC}} + E_{\text{PC}}) \tag{9}$$

Which we can expand as:

$$Y = PW_{\text{PC}}F_{\text{PC}} + PE_{\text{PC}} \tag{10}$$

Let $PE_{\text{PC}} = E_{\text{combined}}$, we now have:

$$Y = PW_{\text{PC}}F_{\text{PC}} + E_{\text{combined}} \tag{11}$$

We have arrived at a reformulation of the MOFA decomposition. From (5) and (10), we can see that $W_{\text{MOFA}} = PW_{\text{PC}}$. However, this equation holds true when using a complete set of principal components and PCA loadings. Since we filtered the principal components based on a cumulative R^2 threshold prior to performing MOFA, we introduce an additional small error, and therefore, this becomes an approximation. Thus P becomes P_{filtered}, W_{PC} becomes $W_{\text{PC-fil}}$, and we arrive at our starting equation:

$$W_{\text{MOFA}} \approx P_{\text{filtered}}W_{\text{PC-fil}} \tag{1}$$

MARMOT: A tool for JDR model comparison and analysis

There are many tools for joint dimensionality reduction of multi-omics data, many of which have been benchmarked [5], however the suitability of various tools seems to be largely dependent on the data and the downstream analysis being performed.

In our analysis, we found the need for comparing JDR models under different conditions. Here we present MARMOT (**M**odel **A**nalysis and compa**R**ison for **M**ulti-**O**mics **T**ools), an R tool for comparing JDR models in different conditions or with different inputs and performing various downstream analyses. MARMOT builds upon the MOMIX benchmarking pipeline published by Cantini et al. [5], with four JDR tools currently implemented.

MARMOT can be accessed and installed from:
`https://github.com/kuijjeralb/MARMOT`

Supplementary figures

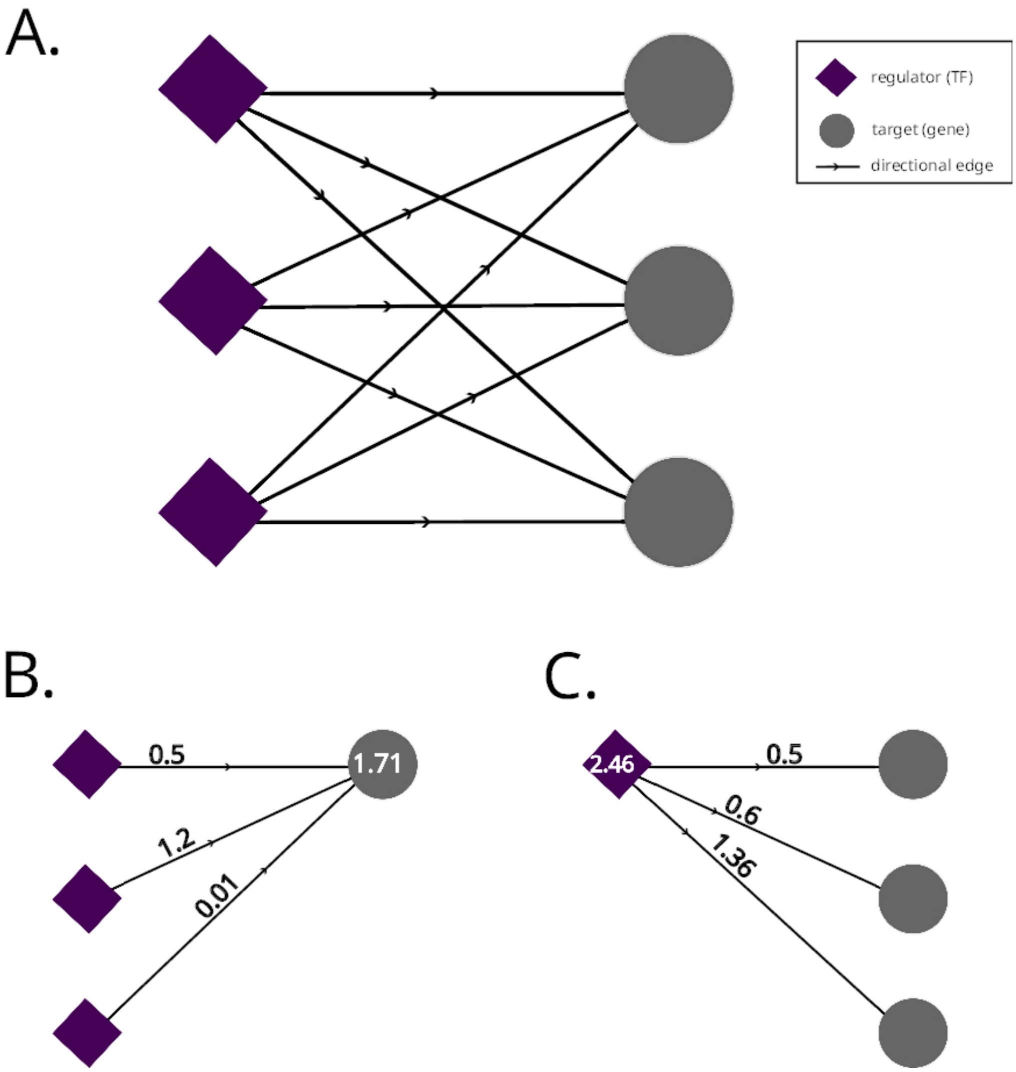

Figure S1. **A.** Illustration of a bi-partite, complete and directional network. **B.** Illustration of the indegree calculation. **C.** Illustration of the outdegree calculation. Alt. text: Three illustrations of networks. The first represents a complete, bi-partite directional network. There are 3 diamond nodes, representing regulators, and three circle nodes, representing genes. Between each TF-gene pair, there is a line with an arrow pointing from the TF to the gene to indicate a directional edge. The second and third illustrations show an example of indegree and outdegree calculations. The same convention applies to represent TFs, genes and edges, with the addition of numbers on the arrows to represent edge weights. The indegree calculation represents one gene with edges coming from three TFs and the sum of the edge weights written in the gene node. The outdegree illustration is the same, but reversed: one TF, three genes.

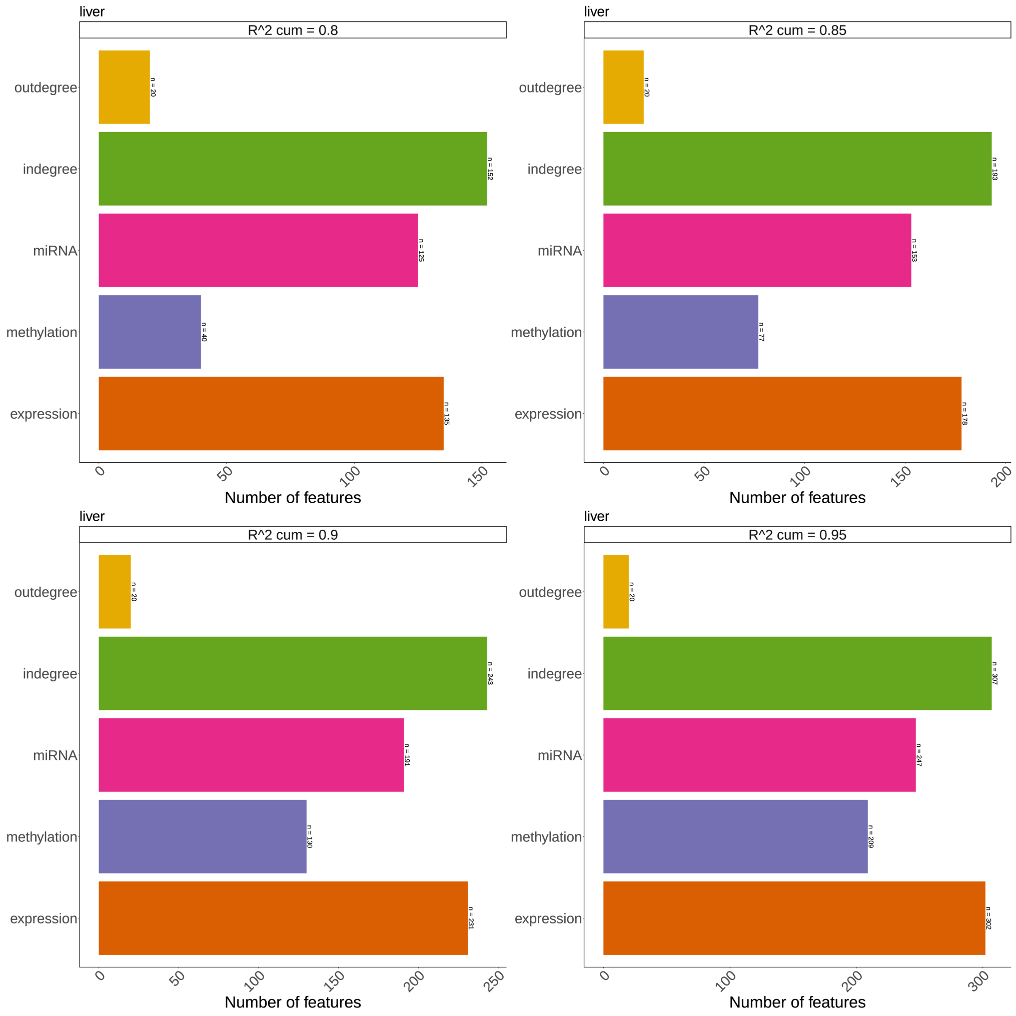

Figure S2. PCA data dimensions when using various cummulative R^2 thresholds in liver cancer. Alt. text: Four barplots showing the number of principal components needed to reach different cummulative R^2 thresholds in liver cancer across omics types. The y axis represents the omics: outdegree, indegree, miRNA and methylation. The x axis represents the number of PCs. The four panels represent four R^2 thresholds: 0.8, 0.85, 0.9 and 0.95 respectively. The minimum number of PCs is 20 in all four panels (outdegree) and the maximum number of PCs is 152, 193. 243 and 307 (indegree) respectively.

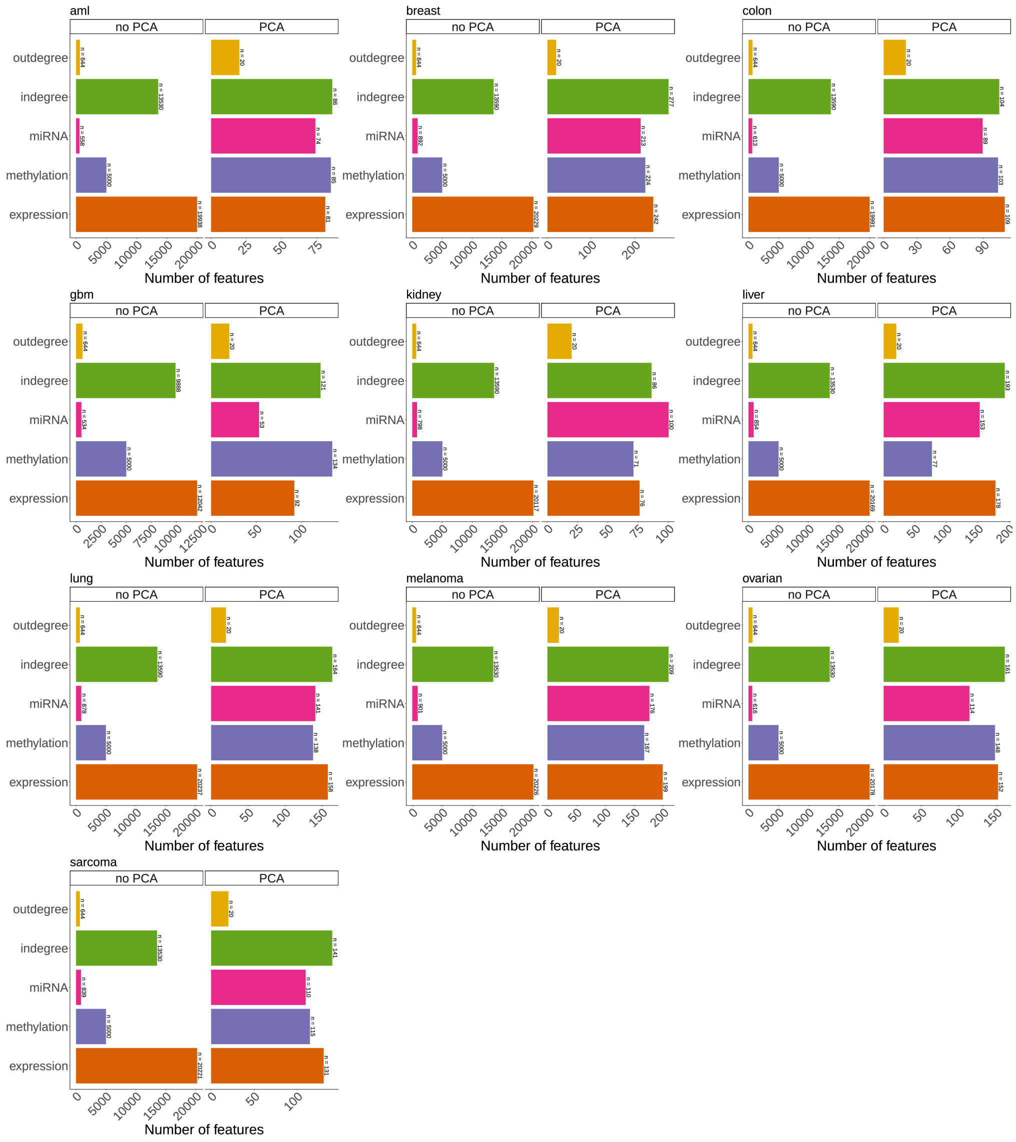

Figure S3. Comparison of data dimensions before and after performing PCA in ten cancer datasets from TCGA. Alt text: Comparison of number of features with and without PCA across all cancer types. This shows similar trends to those described in Figure 2 across all 10 cancer types.

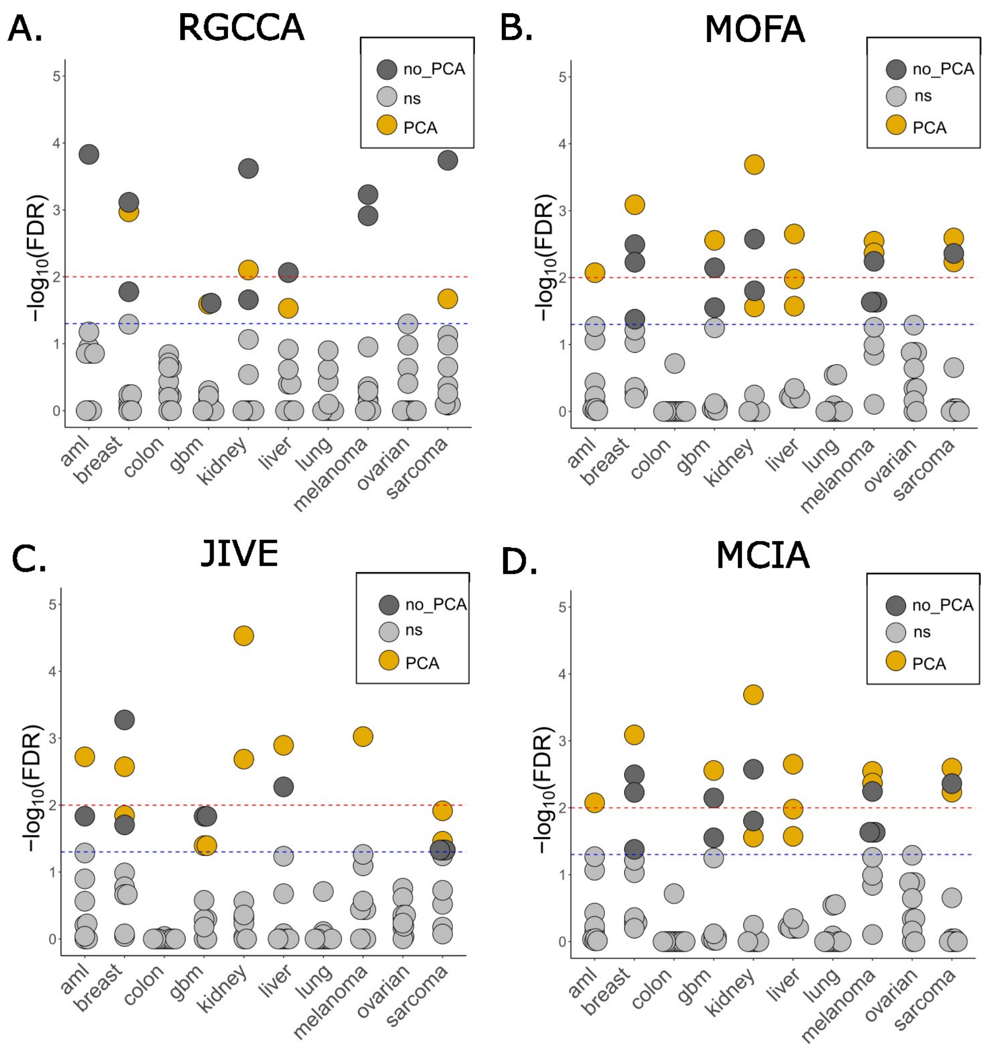

Figure S4. Comparison of factors association with survival with and without PCA in ten cancer datasets from TCGA using four different JDR tools: RGCCA (**A**), MOFA (**B**), JIVE (**C**) and MCIA (**D**). Atl text: Four beeswarm plots showing the -log10(FDR) for the survival association of JDR factors from different models using four different tools: RGCCA, MOFA+, JIVE and MCIA. The x and y axes are the same for all three plots. The x axis lists the different cancer types: AML, breast, colon, GBM, kidney, liver, lung, melanoma, ovarian and sarcoma. The y axis is a -log10 scale of FDR corrected p-values ranging from 0 to 5. Each plot has a significance line drawn at -log10 values of 1.3 and 2. Factors originating from two different models are compared in each plot, one with a the raw data and one with PCA data. The PCA models perform the same, or better when using PCA in three of the four tools: MOFA, JIVE and MCIA. In the case of RGCCA, the PCA models perform notably worse.

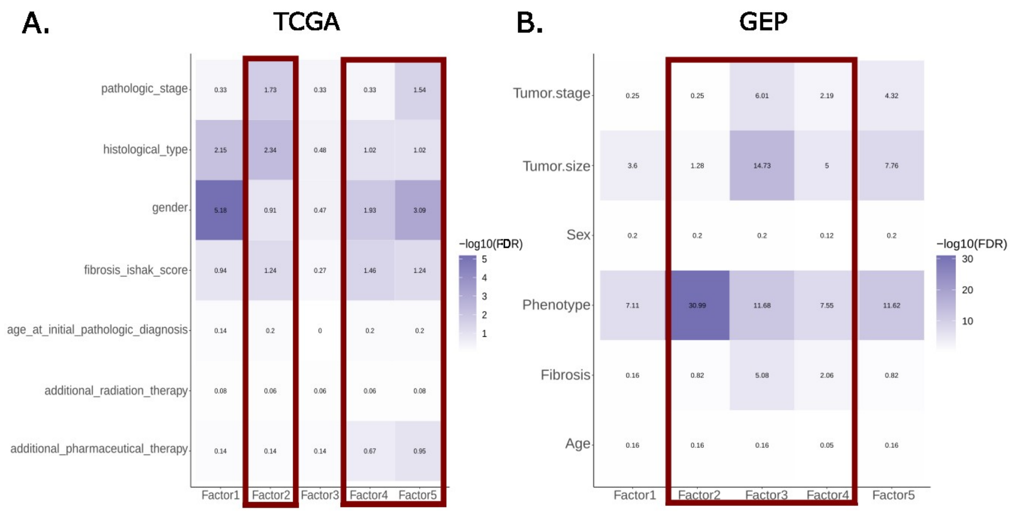

Figure S5. Association of MOFA factors with various clinical features in the TCGA liver (**A**) and GEP liver (**B**) datasets. Highlighted in red are the SAFs. Alt text. Two tile plots showing the association of MOFA+ factor with clinical features in the TCGA and GEP liver datasets. The x axis is the same for both plots, listing he factors from 1 to 5. The y axis lists the clinical features, which are different between the two datasets. In the TCGA dataset, the clinical features are: pathologic stage, histological type, gender, fibrosis ishak score, age at initial pathologic diagnosis, additional radiation therapy and additional pharmaceutical therapy. In the GEP liver dataset, the clinical features are: tumour stage, tumour size, sex, phenotype, fibrosis and age. The tiles are coloured based on the -log10(FDR) of the factor's association with each clinical feature. The survival associated factors in both datasets were not significantly associated with age (FDR ¿ 0.05), while two of the three factors in both datasets were significantly associated with tumour stage (FDR ¡ 0.05). All three survival associated factors were associated with fibrosis in the TCGA dataset, and two in GEP liver. Two of the three survival associated factors were also associated with sex in TCGA, but none in GEP liver.

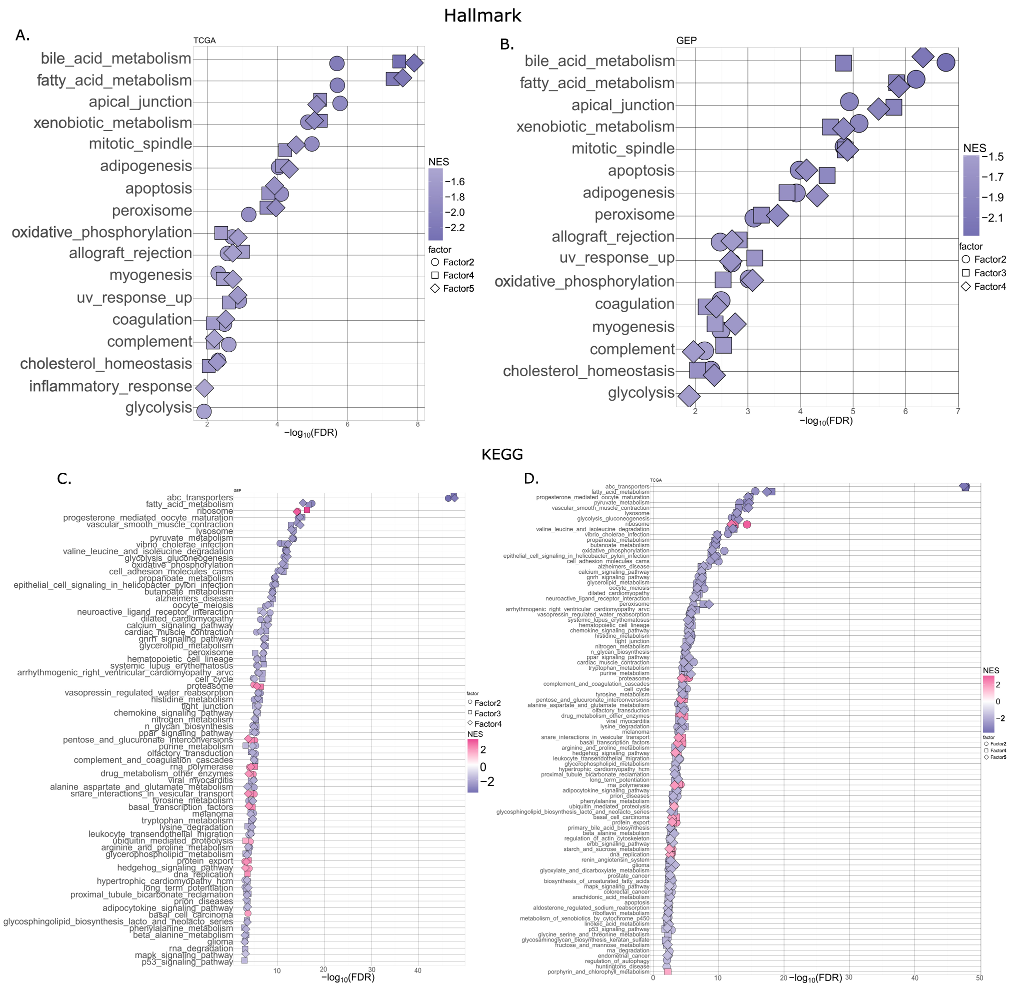

Figure S6. GSEA on indegree factor weights using the Hallmark (**A-B**) and KEGG (**C-D**) gene sets, showing all significant pathways with FDR ¡ 0.01. Alt text: Four bubble plots showing the gene set enrichment of the three survival associated factors in the TCGA and GEP liver cancer datasets. The first two plots show enrichment using the hallmarks gene set and the last two show enrichment using the KEGG gene set. All pathways enriched in the hallmark gene set have a negative normalised enrichment score in both datasets. In both datasets, the top two enriched hallmark pathways are bile acid metabolism and fatty acid metabolism in all three factors. In the KEGG dataset, the top two enriched pathways have a negative normalised enrichment score and are abc transporters and fatty acid metabolism in both datasets across all three factors.

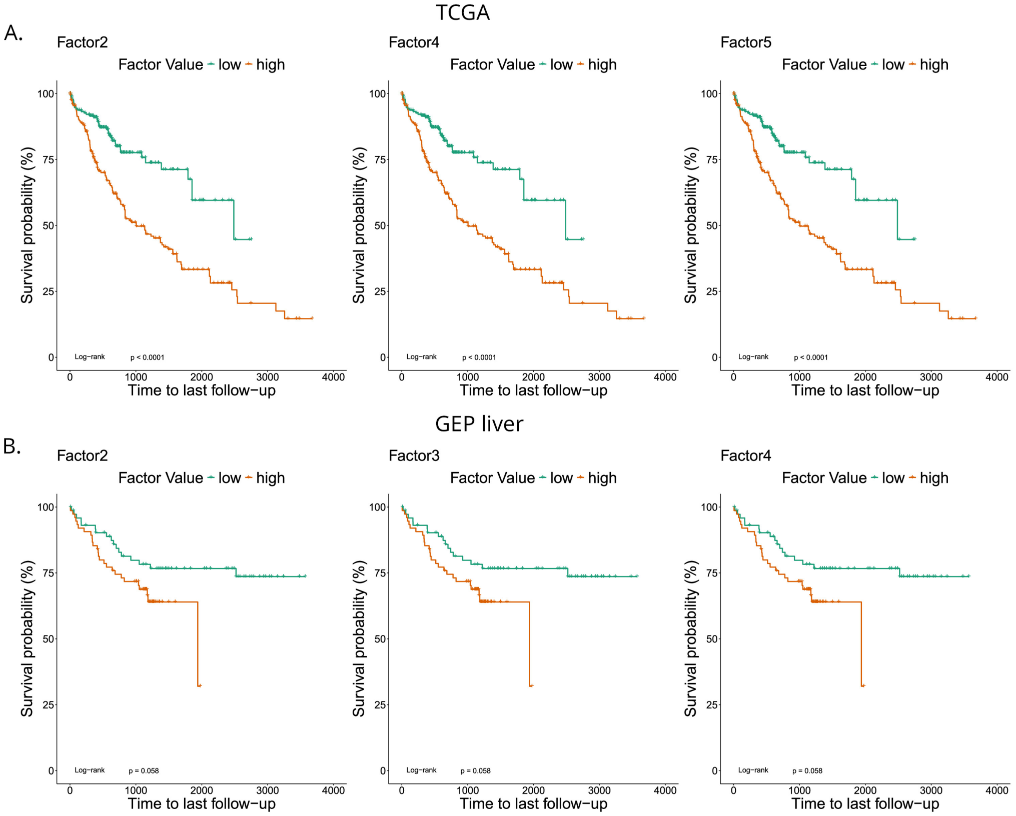

Figure S7. Kaplan-Meier curves when splitting the TCGA (**A**) and GEP (**B**) cohorts based on the median of MOFA survival associated factors. Alt text: Six kaplan meier plots showing the survival differences in patients split by the three survival associated factors in the TCGA and GEP liver datasets. In both datasets, all three factors split the cohorts into the same groups, with low factor values corresponding to better survival and high factor values corresponding to poorer survival.

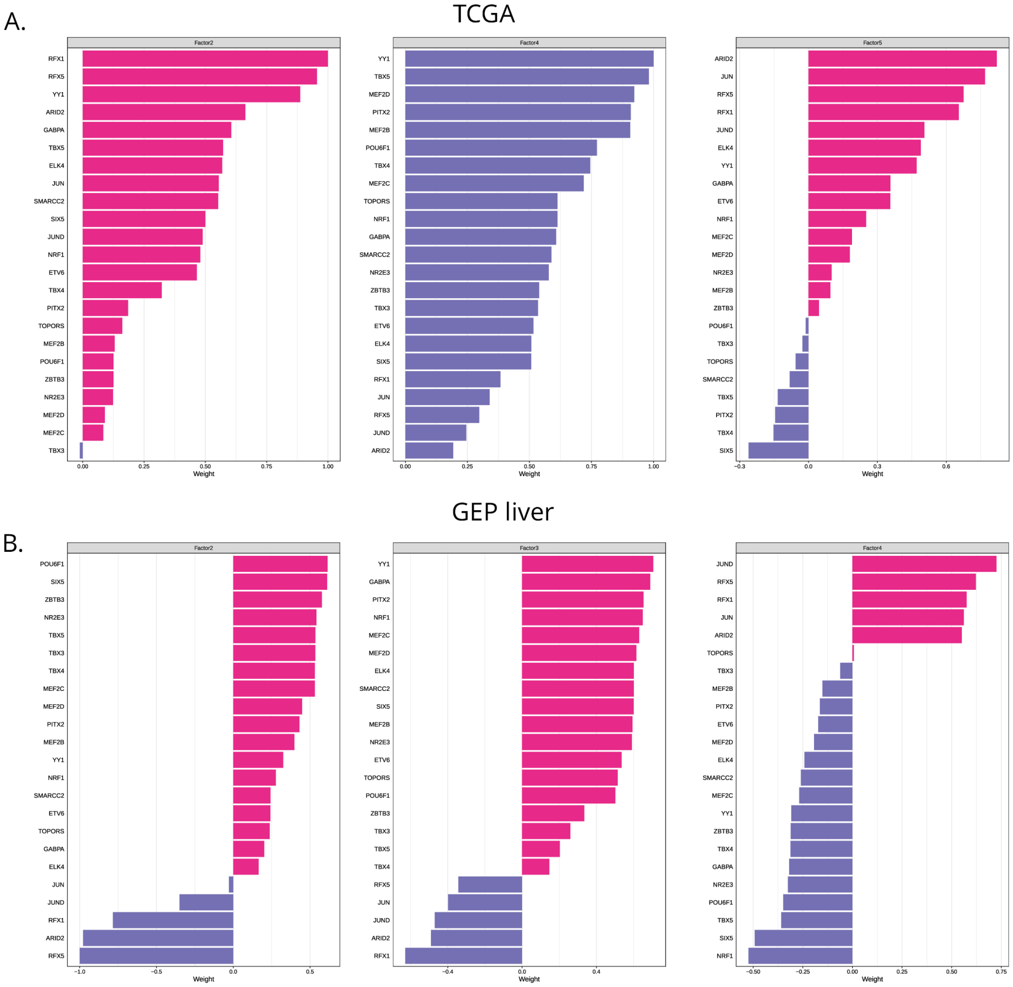

Figure S8. Weights of the 23 TFs shared between the TCGA (A) and GEP (B) datasets when selecting based on a absolute MOFA weight ≥ 0.5 Alt text: Six barplots showing the weights of the 23 shared TFs across the TCGA and GEP liver datasets in each of the three survival associated factors in the two datasets. The x axis represents the factor weights and the y axis lists the 23 TFs: POUF6F1, SIX5, ZBTB3, NR2E3, TBX5, TBX3, TBX4, MEF2C, MEF2D, PITX2, MEF2B, YY1, NRF1, SMARCC2, ETV6, TOPORS, GABPA, ELK4, JUN, JUND, RFX1, ARDI2, RFX5.

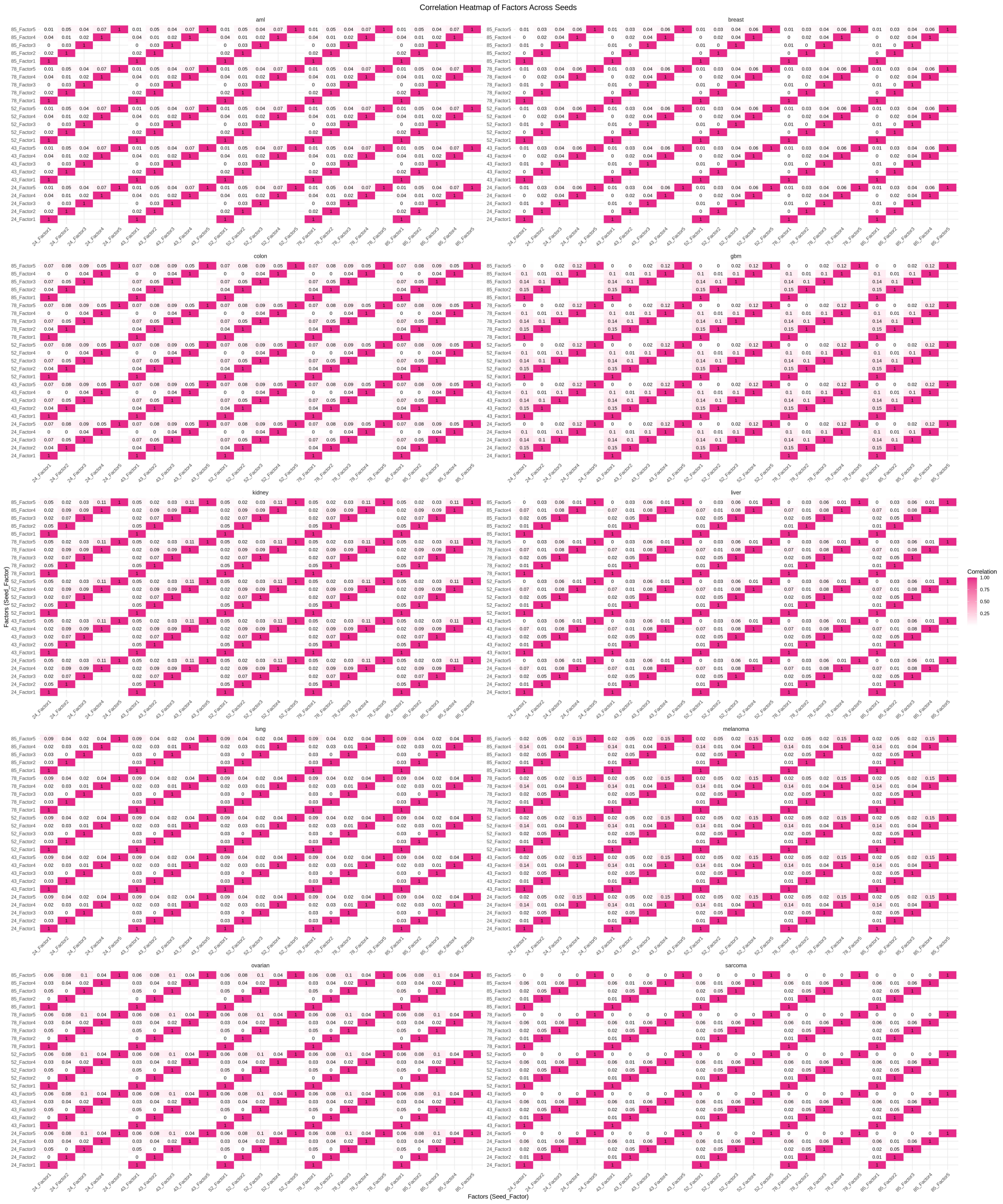

Figure S9. Pairwise Pearson correlation between factors of MOFA models generated with 5 random seed variations. Alt. text: Ten heatmaps showing pairwise pearson correlation between factors of MOFA+ models run with different random seeds in all ten TCGA datasets. The x and y axes are the same, listing the factors from models with 5 different random seeds. The off diagonal correlations in all ten datasets across all factors are 0.1 or less, while the diagonal correlations are all 1.

Supplementary tables

Table S1. Number of samples used for each dataset for GRN inference, for JDR and for survival analysis.

Table S2. Summary of cox regression results of the association of MOFA factors with patient survival when using different seeds in ten TCGA cancer datasets.

Table S3. Summary of cox regression results for the association of MOFA factors with patient survival in a liver cancer dataset from GEPliver.

Table S4. Number of principal components selected for each omic when using different cumulative R^2 thresholds.

Table S5. ELBO scores for MOFA models with different numbers of factors across cancer types.

References

1. Kim D, You K. PCA, SVD, and Centering of Data. arXiv; 2024.
2. Shlens J. A Tutorial on Principal Component Analysis. arXiv; 2014.
3. Argelaguet R, Velten B, Arnol D, Dietrich S, Zenz T, Marioni JC, et al. Multi-Omics Factor Analysis—a Framework for Unsupervised Integration of Multi-omics Data Sets. Molecular Systems Biology. 2018 Jun;14(6):e8124.
4. Argelaguet R, Arnol D, Bredikhin D, Deloro Y, Velten B, Marioni JC, et al. MOFA+: A Statistical Framework for Comprehensive Integration of Multi-Modal Single-Cell Data. Genome Biology. 2020 May;21(1):111.
5. Cantini L, Zakeri P, Hernandez C, Naldi A, Thieffry D, Remy E, et al. Benchmarking Joint Multi-Omics Dimensionality Reduction Approaches for the Study of Cancer. Nat Commun. 2021 Jan;12(1):124.
